# Lutetium-177-PSMA therapy for recurrent/metastatic salivary gland cancer: a prospective pilot study

**DOI:** 10.7150/thno.99035

**Published:** 2024-08-26

**Authors:** Niels J. van Ruitenbeek, Maike J.M. Uijen, Chantal M.L. Driessen, Steffie M.B. Peters, Bastiaan M. Privé, Adriana C.H. van Engen-van Grunsven, Mark W. Konijnenberg, Martin Gotthardt, James Nagarajah, Carla M.L. van Herpen

**Affiliations:** 1 Department of Medical Oncology, Radboud Institute for Health Sciences, Radboud University Medical Center, Nijmegen, The Netherlands.; 2 Department of Medical Imaging, Radboud Institute for Health Sciences, Radboud University Medical Center, Nijmegen, The Netherlands.; 3 Department of Pathology, Radboud Institute for Health Sciences, Radboud University Medical Center, Nijmegen, The Netherlands.; 4 Department of Radiology and Nuclear Medicine, Erasmus Medical Center, Rotterdam, The Netherlands.; 5 Roentgeninstitut Duesseldorf, Duesseldorf, Germany.

**Keywords:** (177)Lu-PSMA therapy, Adenoid cystic carcinoma, Prostate-specific membrane antigen, Salivary duct carcinoma, Salivary gland cancer

## Abstract

There is an urgent need for novel systemic therapies for recurrent/systemic salivary gland cancer, as current treatment options are scarce. [^68^Ga]Ga-PSMA-11 PET/CT revealed relevant uptake of prostate-specific membrane antigen (PSMA) in adenoid cystic carcinoma (AdCC) and salivary duct carcinoma (SDC). Therefore, we assessed the safety, feasibility, efficacy and radiation dosimetry of [^177^Lu]Lu-PSMA-I&T treatment in AdCC and SDC patients in a prospective pilot study.

**Methods:** This single-center, single-arm study intended to include 10 recurrent/metastatic AdCC patients and five recurrent/metastatic SDC patients. AdCC patients could only participate in case of progressive and/or symptomatic disease. Patients required ≥ 1 lesion ≥ 1.5 cm with an SUV_max_ on [^68^Ga]Ga-PSMA-11 PET/CT above liver SUV_mean_. Patients were planned to receive four cycles ~ 7.4 GBq [^177^Lu]Lu-PSMA-I&T. In case of progressive disease per RECIST 1.1 at mid-treatment evaluation after two cycles, treatment was discontinued. Safety was the primary endpoint. Secondary endpoints included objective response rate (ORR), tumor- and organ-absorbed radiation doses and progression-free survival.

**Results:** After screening, 10 out of 15 (67%) AdCC and two out of 10 (20%) SDC patients were eligible. Two patients (17%) demonstrated grade 3 treatment-related toxicity: lymphocytopenia (8%) and hyponatremia (8%). No dose-limiting toxicities occurred. In the AdCC cohort, six patients (60%) completed the four treatment cycles. Due to progressive disease, treatment was discontinued after two cycles in three patients (30%) and after one cycle in one patient (10%). No objective responses were observed (ORR: 0%). Three AdCC patients (30%) showed stable disease ≥ 6 months (7, 17 and 23 months). None of the two SDC patients completed the treatment: one patient deteriorated after the first cycle, while the other had progressive disease after two cycles. The high screen failure rate due to insufficient PSMA uptake resulted in premature closure of the SDC cohort. Dosimetry revealed low tumor-absorbed doses (median 0.07 Gy/GBq, range 0.001-0.63 Gy/GBq).

**Conclusions:** [^177^Lu]Lu-PSMA-I&T in AdCC and SDC patients was safe and generally well-tolerated. However, efficacy was limited, likely due to low tumor-absorbed doses. For SDC, [^177^Lu]Lu-PSMA-I&T appears unfeasible due to insufficient PSMA uptake.

## Introduction

Salivary gland cancer (SGC) is a rare cancer and consists of 22 histological types with distinct clinical behavior and prognosis 1. Among the histological types, recurrent and/or metastatic (R/M) disease most commonly emerges in adenoid cystic carcinoma (AdCC) (60%) and salivary duct carcinoma (SDC) (50%) 2, 3.

AdCC is a secretory gland malignancy that typically arises from the minor salivary glands of the head and neck 4. AdCC is known for its tendency to perineural invasion and hematogenous dissemination 5. Despite aggressive local therapy, most AdCC patients will ultimately develop local recurrences and/or distant metastases 3. Two groups of AdCC patients can be distinguished with different clinical behavior, histomorphology and molecular features: AdCC-1 (37%) and AdCC-2 (63%). AdCC-1 is more aggressive with a poor prognosis (median overall survival [OS] 3.4 years), while AdCC-2 follows an indolent disease course (median OS 23.2 years) 6. In the R/M setting, palliative systemic therapy may be considered in case of objective disease progression and/or new or worsening symptoms that are not otherwise manageable 3, 7. To date, no systemic treatment has been shown to improve OS in R/M AdCC. Platinum-based chemotherapy and multikinase inhibitors resulted in response rates of 13%-25% and 3%-15%, respectively 7, 8. Given the disappointing results of systemic treatments, participation in clinical trials is recommended for these patients 9, 10.

SDC is an aggressive histological type of SGC that most commonly originates from the parotid gland but can also occur in the other salivary glands of the head and neck. Local therapy consists of surgery, which is often combined with lymph node dissection due to high rates of lymph node involvement and is regularly followed by postoperative radiotherapy 9, 10. SDC has a poor prognosis in the R/M setting, with a median OS of five months without anticancer therapy 11. Systemic treatment options include androgen deprivation therapy for androgen receptor-positive SDC (78-96%) and human epidermal growth factor receptor 2 (HER2)-targeted therapies for HER2-positive SDC (29-46%). Androgen deprivation therapy and HER2-targeted therapy resulted in response rates of 42% and 70% and a median OS of 30 and 40 months, respectively 12, 13. Nevertheless, systemic treatment options for this aggressive cancer remain scarce and their efficacy is limited 9, 10.

Prostate-specific membrane antigen (PSMA) is a transmembrane glycoprotein widely recognized as a theranostic target in prostate cancer 14. PSMA-targeted radioligand therapy (PSMA-RLT) proved to be effective for advanced PSMA-positive castration-resistant prostate cancer 17, 18. In the phase 3 VISION trial, [^177^Lu]Lu-PSMA-617 improved the median OS compared to standard care alone (15.3 versus 11.3 months) 19. The beta emitters [^177^Lu]Lu-PSMA-617 and [^177^Lu]Lu-PSMA-I&T are currently the most frequently used therapeutic PSMA tracers in prostate cancer, but have not been compared head-to-head 18, 20.

Based on these remarkable results in prostate cancer, PSMA also gained interest for diagnostic and therapeutic purposes for other cancers 21. The high physiological tracer uptake of the salivary glands on PSMA PET aroused the potential of PSMA theranostics in SGC. After case reports and a case series on positive PSMA immunohistochemistry and [^68^Ga]Ga-PSMA-11 positron emission tomography (PET)/computed tomography (CT) scans in AdCC patients 22-25, our [^68^Ga]Ga-PSMA-11 PET/CT imaging study in 25 SGC patients revealed relevant tumor PSMA uptake in 93% of AdCC cases and 40% of SDC cases 26. This led to the first explorations of PSMA-RLT in SGC patients, with case reports and small cohort studies reporting promising results 27-31.

The urgent need for novel systemic therapies for R/M SGC is evident. With the emergence of PSMA-RLT for prostate cancer, coupled with the identification of the same target in SGC, there is a solid rationale to further explore PSMA-RLT in SGC. Hence, we aimed to investigate the safety, efficacy, feasibility and radiation dosimetry of [^177^Lu]Lu-PSMA-I&T in R/M AdCC and SDC patients in a prospective setting.

## Methods

### Study design

This single-center, single-arm prospective pilot study (NCT04291300) was conducted at the Radboud University Medical Center, an SGC expertise center in The Netherlands. The study consisted of an AdCC cohort and an SDC cohort.

### Patients

Eligible participants were AdCC or SDC patients with incurable, local and/or regional recurrent and/or metastatic disease, aged ≥ 18 years, with adequate bone marrow, renal and liver function, and Eastern Cooperative Oncology Group performance status of 0-2 32. Patients required measurable disease according to Response Evaluation Criteria in Solid Tumors (RECIST) version 1.1 33, which was assessed with baseline tumor imaging consisting of a CT scan of the chest and abdomen and, depending on the primary tumor location, a CT- or magnetic resonance (MR)-scan of the neck. Based on the general indolent tumor growth of AdCC and recommendations for clinical trial design in AdCC patients 7, AdCC patients could only participate in case of objective radiographic disease progression within three months before study enrollment and/or new or worsening disease-related symptoms during the same period that were not otherwise manageable (e.g., bone pain in case of bone metastases).

Screening involved [^68^Ga]Ga-PSMA-11 PET/CT and 2-deoxy-2-[^18^F]fluoro-D-glucose PET/CT, further referred to as [^18^F]FDG PET/CT. Participants required a positive [^68^Ga]Ga-PSMA-11 PET/CT, defined by at least one lesion with a diameter ≥ 1.5 cm with maximum standardized uptake value (SUV_max_) above liver mean standardized uptake value (SUV_mean_). Furthermore, in patients who had several [^18^F]FDG PET/CT positive tumor lesions with low [^68^Ga]Ga-PSMA-11 PET/CT uptake besides the lesion(s) with [^68^Ga]Ga-PSMA-11 PET/CT SUV_max_ above liver SUV_mean_, the eligibility for the study was at the discretion of the treating physicians. Baseline [^68^Ga]Ga-PSMA-11 PET/CT maximum intensity projections of the included patients are added in Figure S1.

Exclusion criteria included pregnancy, inadequate contraceptive measurements for patients with reproductive potential, brain or intracardial metastases, cranial epidural disease, concurrent serious conditions, urinary tract obstruction and an interval of less than four weeks since the last myelosuppressive therapy or other radionuclide therapy.

Complete eligibility criteria are provided in the Supplementary Methods. This study was approved by the Medical Review Ethics Committee Arnhem-Nijmegen, the Netherlands (NL71624.091.19). The study was performed in adherence with the Declaration of Helsinki. All participants provided written informed consent before study enrollment.

### Study procedures

Eligible patients started with the [^177^Lu]Lu-PSMA-I&T treatment within four weeks after the start of screening. The treatment plan consisted of four cycles of 7.4 GBq (±10%) [^177^Lu]Lu-PSMA-I&T, with an interval of six (±1) weeks. [^177^Lu]Lu-PSMA-I&T was administered intravenously over 10 minutes. Premedication for anti-emesis was ensured with ondansetron 8 mg orally one hour before treatment and adequate hydration was advised (approximately two litres of oral fluids on the day of administration and subsequent days). The radiolabeling of [^177^Lu]Lu-PSMA-I&T is described in the Supplementary Methods.

Single-photon emission computed tomography/CT (SPECT/CT) was acquired at five timepoints (1, 24, 48, 72 and 168 hours) after the first [^177^Lu]Lu-PSMA-I&T administration to perform 3D dosimetry. For SPECT/CT imaging, a Siemens Symbia T16 or Intevo Bold gamma camera was used (Siemens Healthineers, Erlangen, Germany). Additional information about image acquisition is outlined in the Supplementary Methods. Blood was collected at nine timepoints (5, 30, 60, 120 and 180 minutes and 24, 48, 72 and 168 hours) postinjection to perform bone marrow dosimetry.

Interim evaluation, i.e. [^68^Ga]Ga-PSMA-11 PET/CT, [^18^F]FDG PET/CT and CT scans, was performed four weeks after the second cycle. In case of progressive disease per RECIST 1.1, the treatment was discontinued. Three months after the fourth cycle, [^68^Ga]Ga-PSMA-11 PET/CT and CT scans were performed, and follow-up including CT scans was continued every three months thereafter.

During study treatment, participants were monitored every two weeks after the first and second treatment cycles and every three weeks after the third and fourth cycles. These visits included blood tests for routine hematology and biochemistry. The final safety assessment was performed six weeks after the fourth cycle.

Patient-reported outcome measures were assessed at baseline, before each treatment cycle and three and six months after the fourth cycle. These included the European Organization for Research and Treatment of Cancer Quality of Life Questionnaire-Core 30 (EORTC-QLQ-C30) 34 and visual analogue scale pain scores.

For all AdCC patients with available formalin-fixed paraffin-embedded tumor tissue, the molecular subtype was determined through P63 immunohistochemical staining, using a cut-off of 10% positive tumor cells 6. An overview of all study assessments (Table S1) and a study flowchart (Figure S2) are included in the Supplementary Materials.

### Dosimetry

Tumors with a diameter > 1 cm were categorized based on the pre-therapeutic [^68^Ga]Ga-PSMA-11 PET/CT SUV_mean_: above, at (±10%) and below liver SUV_mean_. Per tumor site, up to three index lesions per category were selected as index lesions. Organ and tumor dosimetry was performed using Hermes HybridViewer/Dosimetry software (Hermes Medical Solutions, Stockholm, Sweden). Salivary gland and bone lesion volume were determined by average-based iterative thresholding on the baseline [^68^Ga]Ga-PSMA-11 PET/CT images using PMOD 4.4 software (PMOD Technologies, Zurich, Switzerland) 35. The volume of other tumors was determined by a slice-by-slice approach on the baseline CT images, and kidney volume was chosen based on the ICRP89 Male/Female adult model 36. To determine time-integrated activity coefficients, volumes of interest with ~ 1 cm margin were drawn on SPECT/CT images and background correction was applied by drawing a volume of interest near the tumor/organ volume of interest and by subtracting background counts from tumor/organ counts 37. Time-activity curves were fitted to a mono-exponential decay for kidneys and a bi-exponential decay for salivary glands and tumors. Absorbed doses were determined according to the Medical Internal Radiation Dose scheme using Olinda 2.2 (Hermes Medical Solutions, Stockholm, Sweden).

For bone marrow dosimetry, the blood sampling method was used 38. Blood samples were measured in a scintillation counter (248 WIZARD^2^, PerkinElmer, Groningen, The Netherlands) that was calibrated for ^177^Lu to translate from counts per minute to megabecquerels per volume unit (ml). Time-activity curves were fitted to a bi-exponential decay using GraphPad Prism 10.1.2 (GraphPad Software Inc., CA, USA).

### Outcomes

The primary outcome was safety, assessed according to National Cancer Institute's Common Terminology Criteria for Adverse Events version 5.0. Secondary outcomes included objective response rate (ORR), radiation doses absorbed in tumors and organs at risk, progression-free survival (PFS), OS and health-related quality of life (HRQoL). As exploratory endpoints, tumor volumes and the tumor growth rate (TGR) were assessed before and after treatment. ORR was defined as the percentage of patients with a complete or partial response per RECIST 1.1. Tumor- and organ-absorbed radiation doses were calculated as dose per unit activity and as cumulative absorbed dose over all cycles. Cumulative absorbed doses were predicted by extrapolation from cycle 1. PFS was defined as the duration from treatment initiation to progression per RECIST 1.1 or death. OS was defined as the duration from treatment initiation to death. The tumor volume before treatment was determined at baseline, and the volume after treatment was measured three months after cycle 4, or after cycle 2 in case of progression at interim evaluation. Volumes of bone lesions were determined by average-based iterative thresholding on the [^68^Ga]Ga-PSMA-11 PET/CT images, and volumes of other lesions was assessed by a slice-by-slice approach on the CT images. TGR was defined as the percentage change in tumor volume over one month (%/month). TGR was calculated by the formula TGR = 100 × (exp(tumor growth) - 1), where tumor growth = 3 × log(D2/D1)/time (months) 39-41. Tumor size (D) was determined by the sum of the longest diameters of target lesions according to RECIST 1.1. Non-target and new lesions were excluded. For TGR before treatment, D1 represents the tumor size at baseline CT evaluation, and D2 represents the tumor size at the latest CT evaluation at least six months pre-treatment. For TGR after treatment, D1 represents the tumor size at the earliest CT evaluation at least six months after treatment initiation, and D2 represents the tumor size at baseline CT evaluation. TGR was determined only in patients with CT imaging available at least six months before and six months after treatment initiation, while not on other anticancer therapy, so that the growth rate before and after treatment could be compared in these patients.

### Statistical analysis

Because of the explorative nature of this pilot study, no sample size calculation was performed. Considering the rarity of AdCC and SDC, our research team determined that a sample size comprising 10 AdCC and 5 SDC patients would be both feasible and adequate for conducting exploratory analyses. Time-to-event outcomes were analyzed using Kaplan-Meier statistics. Correlations between tumor lesion [^68^Ga]Ga-PSMA-11 PET SUV_mean_ and [^177^Lu]Lu-PSMA-I&T SPECT tumor-absorbed doses, and between tumor-absorbed doses and tumor volume change, were calculated and given as Spearman's r and p-value. A p-value < 0.05 was considered statistically significant. Analyses were performed using R studio version 1.1.463.

## Results

### Patients

Between June 2020 and October 2022, 15 AdCC patients and 10 SDC patients were screened. In the AdCC cohort, 10 out of 15 (67%) screened patients were eligible and enrolled. Ineligibility was due to insufficient [^68^Ga]Ga-PSMA-11 uptake (n = 3) and brain metastases on [^68^Ga]Ga-PSMA-11 PET/CT (n = 2) (Figure S3). All enrolled AdCC patients had objective radiographic disease progression within three months before study enrollment. Among the 10 enrolled AdCC patients, five were male, with a mean age of 61 (range 51-70) years. Eight patients (80%) had the AdCC-2 molecular subtype. All 10 AdCC patients had undergone prior external beam radiation therapy, with 8 patients (80%) who received postoperative radiotherapy in the head/neck area. Two patients (20%) had undergone prior systemic therapy. None of the patients had received prior radionuclide therapy. One AdCC patient (10%) had incurable locoregional disease only, while nine patients (90%) had distant metastases, among whom one patient (10%) also had locoregional recurrence (Table 1).

Two out of 10 (20%) screened SDC patients were eligible and enrolled. Reasons for screen failures were insufficient [^68^Ga]Ga-PSMA-11 uptake (n = 7) and brain metastases on [^68^Ga]Ga-PSMA-11 PET/CT (n = 1). Both enrolled SDC participants were male, ages 64 and 74 years. One SDC patient had undergone prior palliative external beam radiation therapy, and both SDC patients had undergone prior systemic treatment. None of the patients had received prior radionuclide therapy. Both SDC patients had distant metastases, with one also having cutaneous lymphangitis carcinomatosa. The SDC cohort was closed before achieving the intended sample size because most SDC patients were ineligible, mainly due to insufficient PSMA uptake.

The median administered [^177^Lu]Lu-PSMA-I&T activity per cycle was 7.4 (range 6.8-7.5) GBq, with a median of 3 (range 1-4) cycles.

### Safety

**Figure 1:**
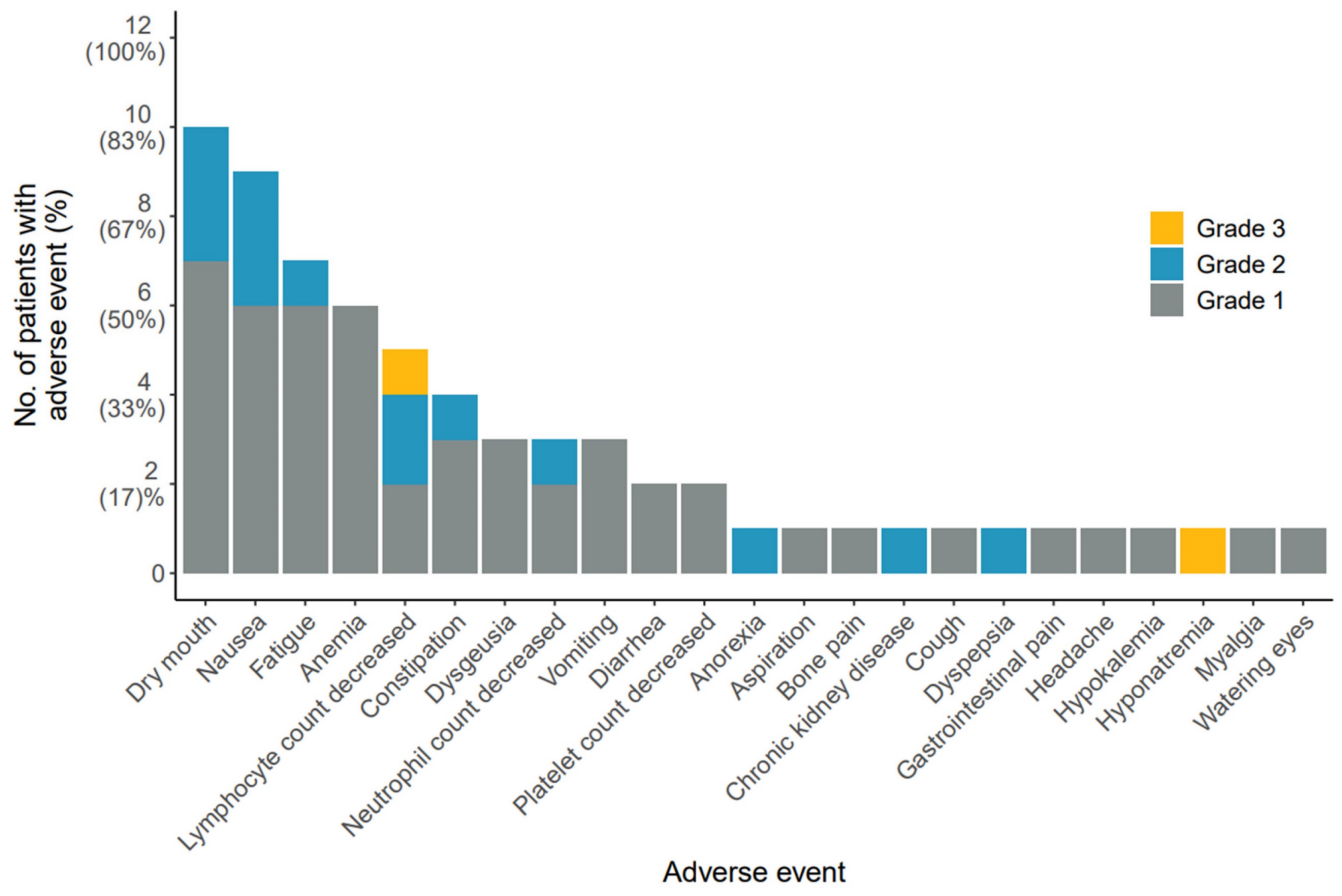
** Treatment-related adverse events.** Figure lists all adverse events possibly, probably, or definitely related to [^177^Lu]Lu-PSMA-I&T treatment, sorted based on frequency of occurrence. Adverse events were graded according to National Cancer Institute Common Terminology Criteria for Adverse Events (version 5.0). Patients were counted once at the highest grade for each adverse event. Abbreviations: ^177^Lu: lutetium-177; PSMA: prostate-specific membrane antigen.

Safety was evaluated for the AdCC and SDC cohorts collectively. No immediate adverse reactions were observed. Common grade 1 or 2 treatment-related adverse events included dry mouth (83%), nausea (75%), fatigue (58%) and anemia (50%). Two patients (17%) demonstrated grade 3 toxicity: one patient developed grade 3 lymphocytopenia after the first cycle, while another patient showed grade 3 hyponatremia following the second cycle. No grade ≥ 4 toxicity was observed (Figure 1). None of the toxicities was dose-limiting. A detailed overview of all adverse events is provided in Table S2.

Noteworthy, one patient developed grade 2 chronic kidney disease four months after the fourth cycle. The estimated glomerular filtration rate, calculated according to the Chronic Kidney Disease Epidemiology Collaboration equitation, gradually decreased from > 90 ml/min/1.73 m^2^ at baseline to a nadir of 32 ml/min/1.73 m^2^ 15 months post-treatment. A kidney biopsy indicated thrombotic microangiopathy, likely attributed to radiation nephropathy. Subsequently, the estimated glomerular filtration rate gradually improved without intervention, until 39 ml/min/1.73 m^2^ 25 months post-treatment.

### Efficacy

**Figure 2:**
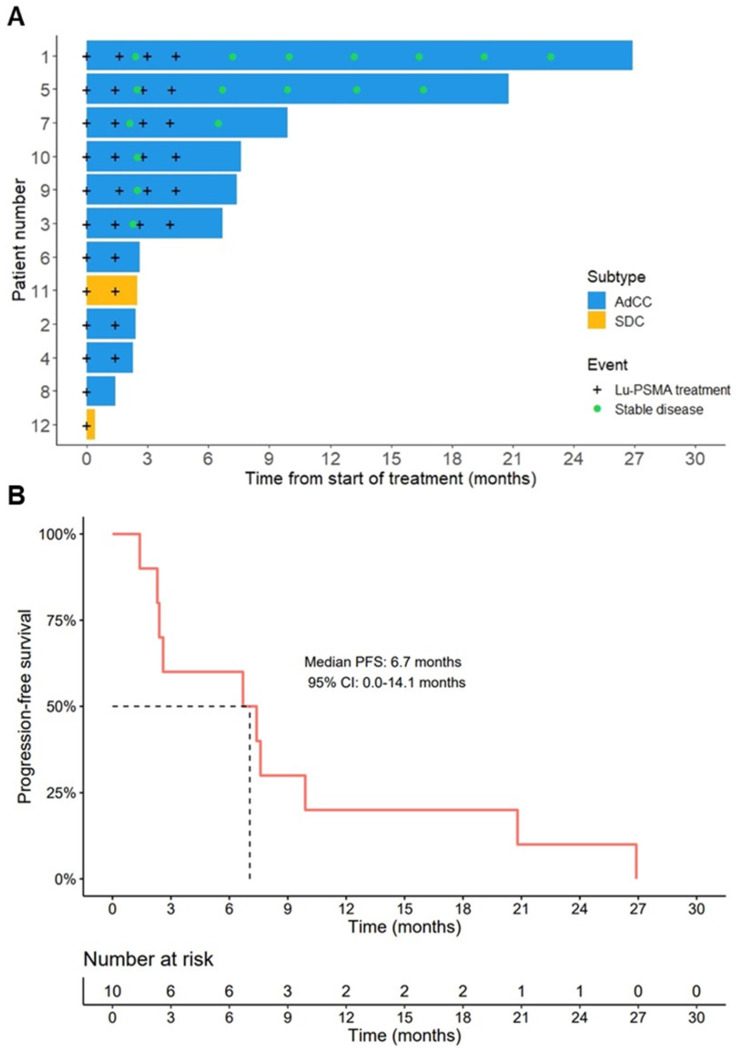
** Efficacy outcomes. (A)** Swimmer plot displaying the time from start of therapy to progressive disease or death (bars), and timepoints of [^177^Lu]Lu-PSMA-I&T treatments and confirmed stable disease (signs). **(B)** Kaplan-Meier estimate of progression-free survival of the AdCC cohort. Abbreviations: ^177^Lu: lutetium-177; AdCC: adenoid cystic carcinoma; 95% CI: 95% confidence interval; Lu: lutetium-177; PFS: progression-free survival; PSMA: prostate-specific membrane antigen; SDC: salivary duct carcinoma.

**Figure 3:**
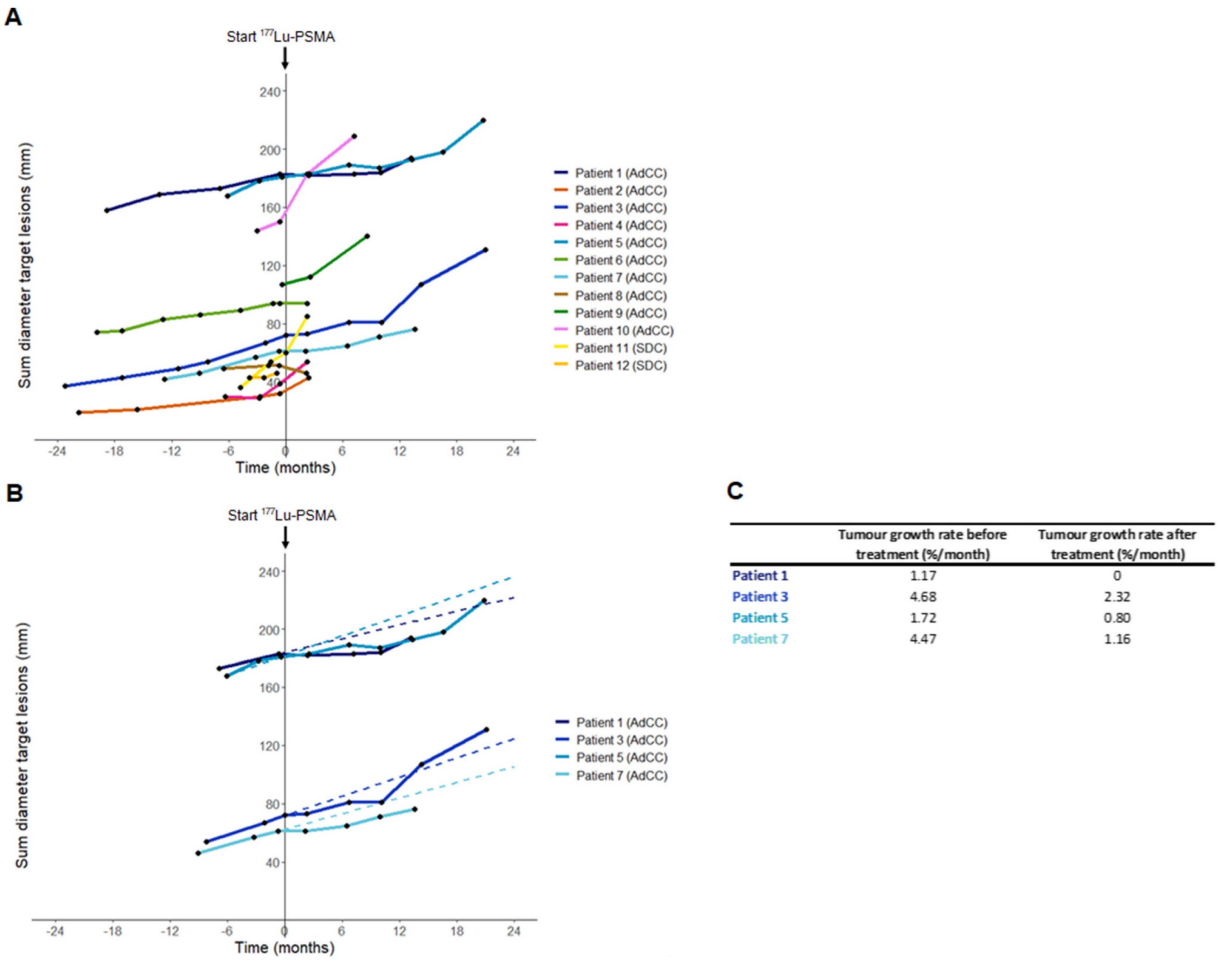
** Sum diameter of RECIST target lesions and tumor growth rate before and after [^177^Lu]Lu-PSMA-I&T treatment. (A)** Sum diameter of RECIST target lesions of all patients. T=0 indicates the start of [^177^Lu]Lu-PSMA-I&T therapy. Only imaging timepoints where patients were not on other anticancer therapy are visualized. **(B)** Sum diameter of RECIST target lesions of patients with CT imaging available both at least six months before and six months after treatment initiation. Pre-therapy timepoints are only visualized from the latest timepoint at least six months pre-treatment. Dashed lines represent the tumor growth rate before treatment. Only imaging timepoints where patients were not on anticancer therapy are visualized. **(C)** Tumor growth rate before and after treatment of patients with imaging available both at least six months before and six months after treatment initiation. Abbreviations: ^177^Lu: lutetium-177; AdCC: Adenoid cystic carcinoma; CT: computed tomography; PSMA: prostate-specific membrane antigen; RECIST: Response Evaluation Criteria in Solid Tumors; SDC: salivary duct carcinoma.

**Figure 4:**
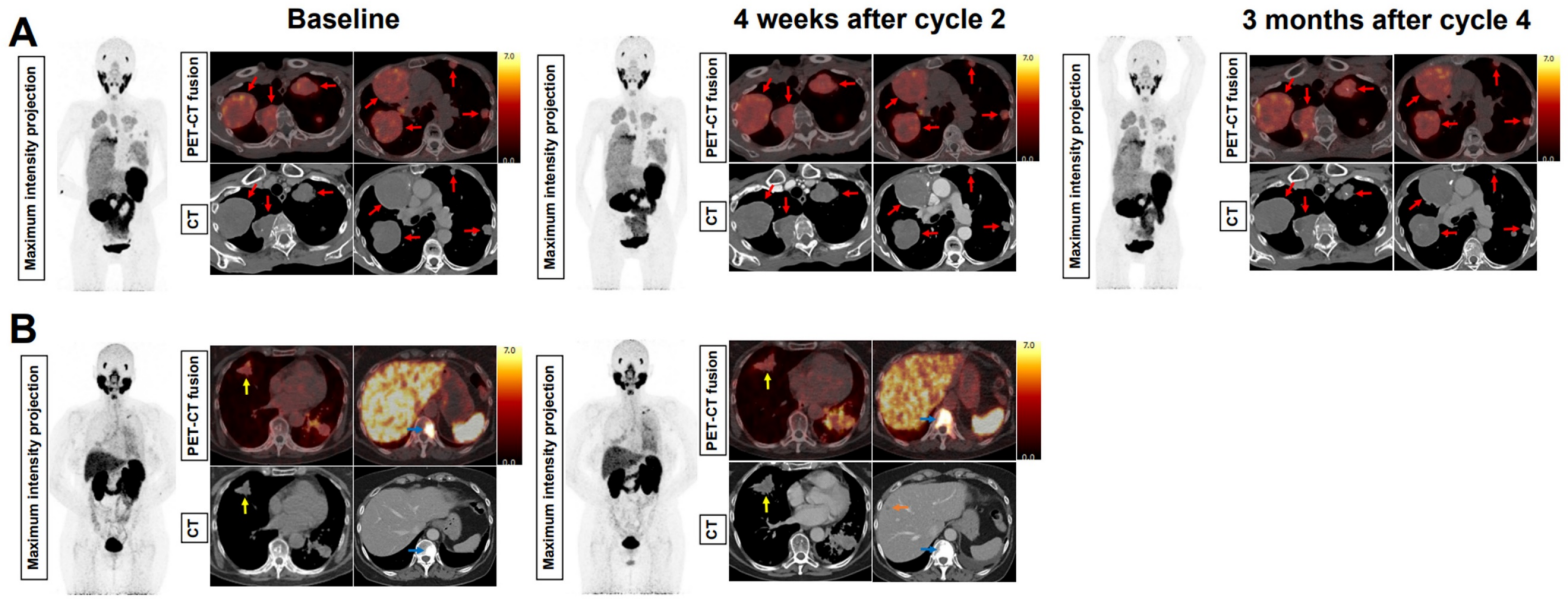
** Baseline and evaluation PSMA-PET and CT images of representative cases with stable disease and progressive disease. (A)** 70-year old woman with AdCC originating from Bartholin's gland (status post surgery), with metastases in the lungs and the pleurae. At interim evaluation four weeks after the second [^177^Lu]Lu-PSMA-I&T treatment cycle and at evaluation three months after the fourth treatment cycle, the size of all metastases was stable (red arrows). The patient had stable disease until 23 months after treatment initiation. **(B)** 63-year old woman with AdCC originating from Bartholin's gland (status post surgery), with metastases in the lungs, the liver, and the bones. At interim evaluation after the second [^177^Lu]Lu-PSMA-I&T treatment cycle, progressive disease was observed. This included growth of the lung metastases (yellow arrows) and the bone metastases (white arrows), and a new liver metastasis (orange arrow). Radioligand therapy was ceased due to the progression. Abbreviations: ^177^Lu: lutetium-177; AdCC: Adenoid cystic carcinoma; CT: computed tomography; PET, positron emission tomography; PSMA: prostate-specific membrane antigen.

In the AdCC cohort, six patients (60%) received the four planned treatment cycles. Treatment was discontinued at interim evaluation after two cycles in three AdCC patients (30%) due to progressive disease. Therapy was ceased in one AdCC patient (10%) after one cycle due to the occurrence of a pathological vertebral fracture causing spinal cord compression, which was associated with radiological evidence of disease progression. No objective responses were observed during the trial (ORR: 0%). At evaluation three months after completion of four treatment cycles, three patients showed progressive disease, while three patients had stable disease. Their duration of disease stability extended until 7, 17 and 23 months (Figure 2A). With a median follow-up of 19.2 months (interquartile range 12.5-27.0 months) in the AdCC cohort, the median PFS was 6.7 months (95% CI 0.0-14.1 months) (Figure 2B), and the median OS was 27.0 months (95% CI 10.1-44.0 months). The TGR before and after treatment could be determined in four patients. In these patients, the TGR after treatment appeared to be lower compared to the TGR before treatment (Figure 3A-C). In the other patients, the TGR could not be determined due to missing imaging timepoints. Images of representative cases with stable disease and with progressive disease are displayed in Figure 4A and 4B, respectively.

In the SDC cohort, treatment was discontinued after one treatment cycle in one patient due to rapid clinical deterioration, leading to death two weeks after treatment initiation. The other patient ceased treatment after disease progression at interim evaluation after two cycles, and he deceased five months later (Figure 2A).

### Dosimetry

**Figure 5:**
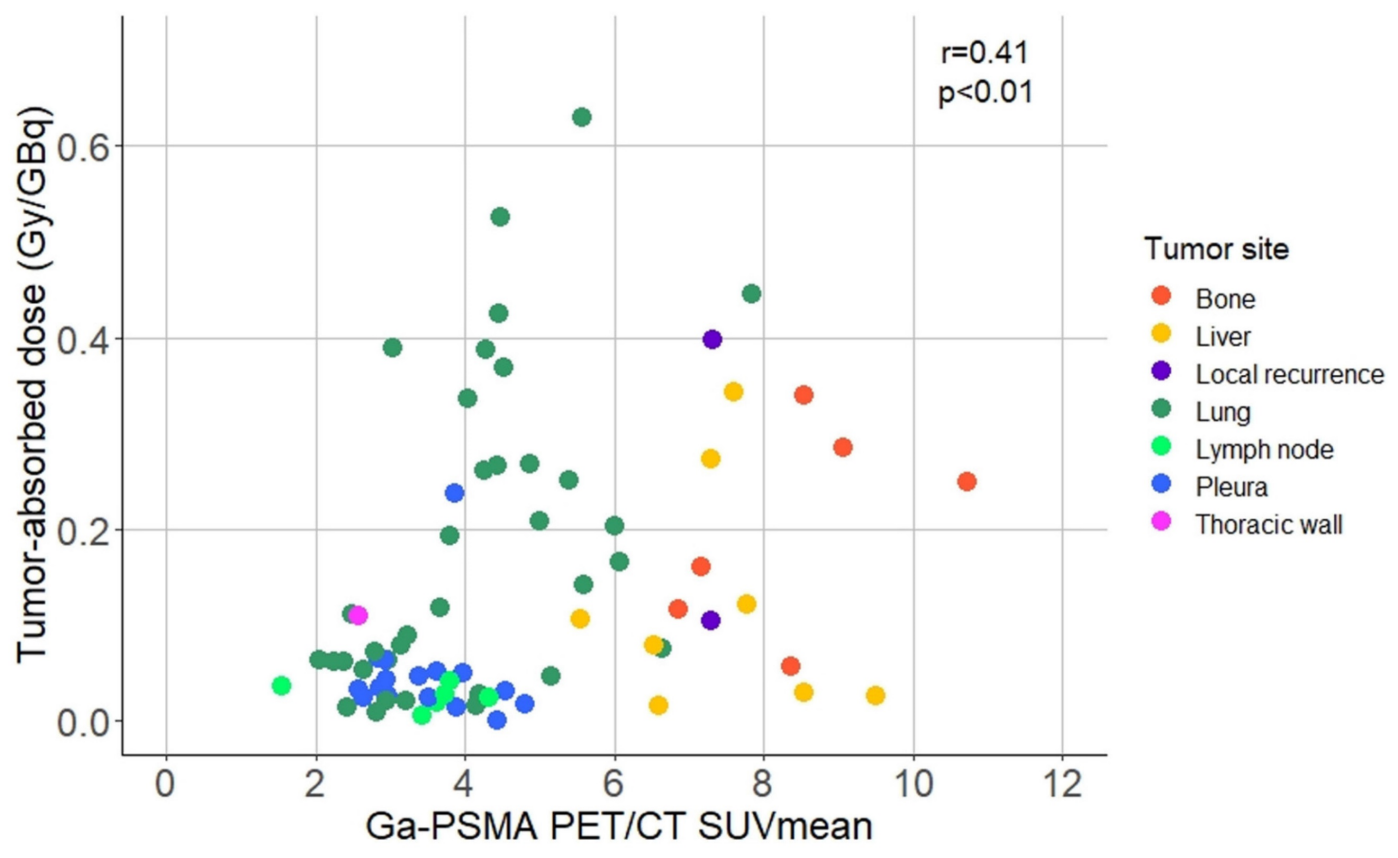
** Scatter plot displaying the correlation between the baseline [^68^Ga]Ga-PSMA-11 PET/CT SUV_mean_ and the absorbed dose per index tumor lesion.** Abbreviations: ^68^Ga: gallium-68; CT: computed tomography; Ga: gallium-68; PET: positron emission tomography; PSMA: prostate-specific membrane antigen; SUVmean: mean standardized uptake value.

Dosimetry was performed in all patients, except for the SDC patient who rapidly deteriorated after the first treatment cycle. Patients had between one and eleven tumor lesions that were assessed by dosimetry. The median tumor-absorbed dose in the AdCC cohort was 0.06 Gy/GBq (range 0.001-0.63 Gy/GBq), and 0.14 Gy/GBq (range 0.01-0.34 Gy/GBq) in the SDC patient (Table 2A). The average pre-therapeutic tumor [^68^Ga]Ga-PSMA-11 PET/CT SUV_mean_ was 4.71±2.04. Higher tumor-absorbed doses correlated with higher pre-therapeutic [^68^Ga]Ga-PSMA-11 PET/CT SUV_mean_ (r = 0.41; p < 0.01) (Figure 5). Tumor-absorbed doses did not significantly correlate with the tumor volume change after therapy compared to baseline (r = 0.19, p = 0.098) (Figure S4). Organ dosimetry was evaluated for the AdCC and SDC cohorts collectively. The mean kidney, salivary gland and bone marrow absorbed doses were 0.82±0.31 Gy/GBq, 0.74±0.36 Gy/GBq and 0.007±0.002 Gy/GBq, respectively (Table 2B). Detailed dosimetry results of tumor lesions and organs at risk are provided in Table S3 and Table S4, respectively.

### Patient-reported outcome measures

HRQoL scores did not significantly change during the treatment period. Two patients experienced short-term improvement in VAS pain scores after the first treatment cycle, but these did not persist over time (Figure S5).

## Discussion

This is the first prospective study that evaluated the safety, efficacy, feasibility and radiation dosimetry of [^177^Lu]Lu-PSMA-I&T radioligand therapy in R/M SGC. Overall, the treatment was well-tolerated, and no dose-limiting toxicities occurred. In the 10 treated AdCC and 2 SDC patients no objective responses were observed. Three AdCC patients demonstrated durable stable disease of 7, 17 and 23 months. For SDC, [^177^Lu]Lu-PSMA-I&T therapy was deemed unfeasible due to the high screen failure rate attributed to low and heterogeneous PSMA expression, resulting in premature closure of the SDC cohort.

The toxicity profile observed in this SGC population resembled that of [^177^Lu]Lu-PSMA studies in prostate cancer and organ-absorbed doses were similar 42. However, we observed two clinically relevant differences. First, we observed a lower grade ≥ 3 hematological toxicity rate (8% lymphocytopenia only) compared to end-stage prostate cancer patients in the VISION trial (13% anemia, 8% lymphocytopenia, 8% thrombocytopenia and 3% leukopenia). This difference might be explained by the compromised bone marrow in end-stage prostate cancer, due to prior myelosuppressive systemic treatments (99% previous taxane therapy) and extensive bone metastases (91% bone metastases) in most prostate cancer patients 19. Within our SGC population, fewer patients had prior myelosuppressive systemic therapy (25%) and bone metastases (33%).

Second, the xerostomia rate in the present study was relatively high compared to prostate cancer patients (83% versus 39%, respectively) 43. It is clinically relevant to note that the rate of grade 2 xerostomia in the present study was also significantly higher than in prostate cancer (25% versus 14%, respectively) 44. Most SGC patients received prior radiotherapy and/or surgery in the head-and-neck region, resulting in xerostomia at baseline. The lower salivary gland tissue capacity makes this population susceptible to further decline in salivary gland function after PSMA-RLT. Within our study, the increase in xerostomia was reversible in all cases, consistent with observations from prostate cancer studies.

Of particular interest, one patient developed grade 2 chronic kidney disease four months after her fourth [^177^Lu]Lu-PSMA-I&T treatment cycle. A recent case series reported three similar cases of chronic kidney disease following [^177^Lu]Lu-PSMA-I&T, associated with radiation nephropathy 45. The cumulative kidney-absorbed dose our patient received was lower (24.5 Gy) compared to the three patients described in this series (range 39.5-50.0 Gy). As reported previously, kidney-absorbed doses with [^177^Lu]Lu-PSMA-I&T are ~ 1.5x higher than with [^177^Lu]Lu-PSMA-617, potentially increasing the risk of nephropathy 46.

In the present study and the prior literature together, a total of 23 AdCC patients and 3 SDC patients treated with [^177^Lu]Lu-PSMA have been described, yet no objective responses of [^177^Lu]Lu-PSMA have been reported in any of these patients. A recent study evaluated [^177^Lu]Lu-EB-PSMA-617, which is [^177^Lu]Lu-PSMA-617 conjugated with the albumin binder Evans Blue to decelerate plasma clearance. Symptoms of all four patients in this study improved; nevertheless, objective response per RECIST was not reported. The other studies were case series and case reports 27, 28, 30, 31. Objective responses were not observed in any of these papers, while symptom improvement and stable disease were described. However, caution is warranted when attributing stable disease to a treatment effect in AdCC, especially considering that 80% of the AdCC patients in our study had the indolent AdCC-2 molecular subtype, although progressive and/or symptomatic disease was a prerequisite for inclusion in this study 6, 7. Clearly, the efficacy of [^177^Lu]Lu-PSMA in SGC patients is significantly less pronounced compared to prostate cancer patients, where objective responses were reported in 51% of RECIST-evaluable patients 19.

In the present study, we conducted extensive state-of-the-art 3D SPECT/CT dosimetry which revealed low tumor-absorbed doses, possibly explaining the lack of efficacy of [^177^Lu]Lu-PSMA-I&T in SGC patients. Median tumor-absorbed doses were only 0.06 Gy/GBq in the AdCC cohort and 0.14 Gy/GBq in the SDC cohort. Two prior studies on PSMA-RLT in SGC that performed dosimetry have been published to date, both employing [^177^Lu]Lu-PSMA-617. These studies showed slightly higher doses than our study (AdCC mean 0.41 Gy/GBq; SDC range 0.06-0.68 Gy/GBq) 30, 31. Still, these doses are substantially lower compared to [^177^Lu]Lu-PSMA-I&T studies in prostate cancer (median tumor-absorbed dose 3.3 Gy/GBq) 47, 48. We propose two potential causes for the low tumor-absorbed doses in the present study. First, the PSMA expression in SGC appears lower and more heterogeneous, with [^68^Ga]Ga-PSMA-11 uptake generally being lower in SGC (average SUV_max_ 8.2 in the present study) compared to prostate cancer (average SUV_max_ 15.8-23.2) 49. Second, patient selection in current prostate cancer guidelines is more stringent than in our study, not allowing a relevant fraction of PSMA-negative tumors 50.

Based on the current study, selection for PSMA-RLT with [^68^Ga]Ga-PSMA-11 PET/CT appears insufficient in predicting tumor doses for SGC patients. A more reliable selection procedure is warranted for this population. Pre-therapeutic tumor dosimetry, such as with zirconium-89 labeled PSMA, could be an effective approach 52. This technique allows for calculating the doses in tumors and healthy organs prior to therapy, which also enables adjustments in the administered radioligand activity tailored to each patient. Following proper selection, PSMA-RLT with only a beta emitter may still be inadequate to achieve a sufficient therapeutic effect. The use of alpha emitters such as actinium-225 may be more effective, as alpha particles are much more energetic and can induce double-stranded DNA-breaks 53. In contrast to other non-prostate cancer types, PSMA is predominantly expressed on AdCC tumor cells rather than on the neovasculature 21. Therefore, the shorter tissue penetration range of alpha emitters should not be a limiting factor in AdCC. The high rate of xerostomia with alpha emitters - 91% after three cycles in prostate cancer - yet could be a limiting factor in the SGC population with a propensity to decline in salivary gland function 53. To minimize xerostomia while enhancing the efficacy, “tandem” therapy with low-activity actinium-225 and full activity [^177^Lu]Lu-PSMA may be considered 54.

Limitations of this study include its relatively small sample size and single-arm design, although these are common characteristics in studies addressing this rare disease. Furthermore, dosimetry was conducted only at the first therapy cycle, with cumulative absorbed doses extrapolated from this cycle. Recent data however indicated that extrapolation of dosimetry from cycle 1 is reliable 55. Last, the tumor growth before and after treatment is not a validated outcome measure and could only be assessed in a minority of patients, so these results should be considered exploratory.

## Conclusion

We conclude that [^177^Lu]Lu-PSMA-I&T for R/M AdCC and SDC patients exhibits an acceptable toxicity profile. However, the clinical benefit observed in these patients was limited, likely due to low tumor-absorbed doses. While PSMA-RLT might be feasible for a proportion of AdCC with improved patient selection and individualization of the treatment plan, it appears unfeasible for SDC due to the predominantly insufficient PSMA-ligand uptake.

## Supplementary Material

Supplementary methods, figures and tables.

## Figures and Tables

**Table 1:** Patient characteristics.

Characteristic	AdCC (n=10)	SDC (n=2)	
Age, median, years (range)	61 (51-70)	69 (64-74)	
Sex, No. (%)			
Female	5 (50)	0	
Male	5 (50)	2 (100)	
ECOG performance status, No. (%)			
0	4 (40)	0	
1	5 (50)	2 (100)	
2	1 (10)	0	
Primary site, No. (%)			
Minor salivary gland	4 (40)	0	
Submandibular gland	3 (30)	1 (50)	
Bartholin's gland	2 (20)	0	
Trachea	1 (10)	0	
Lacrimal gland	0	1 (50)	
AdCC molecular subtype,^a^ No. (%)			
AdCC-1	1 (10)	N/A	
AdCC-2	8 (80)	N/A	
Unknown	1 (10)	N/A	
Disease distribution, No. (%)			
Metastatic disease	8 (80)	2 (100)	
Locoregional and metastatic disease	1 (10)	0	
Locoregional disease	1 (10)	0	
Metastasis sites,^b^ No. (%)			
Lung	9 (100)	0	
Pleura	5 (56)	0	
Liver	4 (44)	1 (50)	
Lymph nodes	3 (33)	2 (100)	
Bone	2 (22)	2 (100)	
Other	1 (11)	2 (100)	
Prior surgical resection of the primary tumor, No. (%)	8 (80)	1 (50)	
Prior postoperative radiotherapy, No. (%)	8 (80)	0	
Prior palliative radiotherapy, No. (%)	4 (40)	1 (50)	
Prior systemic radionuclide therapy, No. (%)	0	0	
Prior systemic therapy lines, No. (%)			
0	8 (80)	0	
1	2 (20)	1 (50)	
2	0	0	
3	0	1 (50)	
Time from initial diagnosis to PSMA-RLT, median, months (range)	98 (29-277)	40 (15-64)	
Time from first R/M disease to PSMA-RLT, median, months (range)	25 (3-59)	24 (15-33)	
			

^a^According to Ferrarotto *et al.* 6; ^b^Metastasis site included only patients with distant metastases (AdCC, n=9; SDC, n=2); Abbreviations: AdCC: adenoid cystic carcinoma; ECOG: Eastern Cooperative Oncology Group; N/A: not applicable; PSMA: prostate-specific membrane antigen; PSMA-RLT: PSMA-targeted radioligand therapy; R/M: recurrent and/or metastatic; SDC: salivary duct carcinoma.

**Table 2A:** Dosimetry results; Absorbed dose in index tumor lesions.

Type	Lesion number	Dose per administered activity (Gy/GBq)			Cumulative dose (Gy)		
		Mean (±SD)	Median	Range	Mean (±SD)	Median	Range
**AdCC**	66	0.14 (0.15)	0.06	0.001-0.63	3.39 (4.23)	1.69	0.01-18.67
**SDC**	8	0.16 (0.13)	0.14	0.01-0.34	2.36 (1.96)	2.12	0.08-5.16

AdCC, adenoid cystic carcinoma; SD, standard deviation; SDC, salivary duct carcinoma

**Table 2B:** Dosimetry results; Absorbed dose in organs at risk.

Organ	Dose per administered activity (Gy/GBq)			Cumulative dose (Gy)		
	Mean (±SD)	Median	Range	Mean (±SD)	Median	Range
**Kidneys**	0.82 (0.31)	0.73	0.42-1.34	18.62 (11.56)	15.41	4.97-39.51
**Salivary glands**	0.74 (0.36)	0.69	0.27-1.28	16.45 (10.61)	15.25	4.92-34.07
**Bone marrow**	0.007 (0.002)	0.007	0.003-0.010	0.154 (0.071)	0.135	0.041-0.294

Abbreviations: AdCC: adenoid cystic carcinoma; SD: standard deviation; SDC: salivary duct carcinoma.

## Abbreviations

[^18^F]FDG: 2-deoxy-2-[^18^F]fluoro-D-glucose
AdCC: adenoid cystic carcinoma
CT: computed tomography
EORTC-QLQ-C30: European Organization for Research and Treatment of Cancer Quality of Life Questionnaire-Core 30
HER2: human epidermal growth factor receptor 2
HRQoL: health-related quality of life
MR: magnetic resonance
ORR: objective response rate
OS: overall survival
PET: positron emission tomography
PFS: progression-free survival
PSMA: prostate-specific membrane antigen
PSMA-RLT: PSMA-targeted radioligand therapy
R/M: recurrent and/or metastatic
RECIST: Response Evaluation Criteria in Solid Tumors
SDC: salivary duct carcinoma
SGC: salivary gland cancer
SPECT: Single-photon emission computed tomography
SUV_max_: maximum standardized uptake value
SUV_mean_: mean standardized uptake value
TGR: tumor growth rate
