# Supplementary material for: Lutetium-177-PSMA therapy for recurrent/metastatic salivary gland cancer: a prospective pilot study
Source: Theranostics. 2024 Aug 26;14(14):5388–99. doi: 10.7150/thno.99035 (PMC11413786; doi:10.7150/thno.99035)
Supplement: Supplementary file 1 — Supplementary methods, figures and tables. [file thnov14p5388s1.pdf]

Supplementary Materials

Table of contents

Supplementary Methods **2**

Inclusion and exclusion criteria 2

Labeling and purification of PSMA-I&T with ^{177}Lu 3

Image acquisition and reconstruction 4

Supplementary Tables **5**

Table S1: Study assessments table 5

Table S2: All adverse events (any cause) 6

Table S3: Index tumor lesions [^{68}Ga]Ga-PSMA-11 PET/CT SUV values, tumor-absorbed doses per unit administered activity and cumulative dose (Gy/GBq or Gy), and tumor volumes before and after ^{177}Lu-PSMA therapy. 8

Table S4: Absorbed dose in organs at risk per patient per unit administered activity and cumulative dose (Gy/GBq or Gy) 11

Supplementary Figures **12**

Figure S1: [^{68}Ga]Ga-PSMA-11 PET/CT maximum intensity projections before [^{177}Lu]Lu-PSMA-I&T treatment. 12

Figure S2: Study flowchart 13

Figure S3: Trial profile of the AdCC and the SDC cohort 14

Figure S4: Scatter plot displaying the correlation between the absorbed radiation dose per index tumor lesion and the tumor volume change after [^{177}Lu]Lu-PSMA-I&T therapy compared to baseline. 15

Figure S5: Individual patient-reported outcome measure scores. 16

Supplementary Methods

Inclusion and exclusion criteria

Inclusion criteria:

• Ability to provide written informed consent.
• Age ≥18 years.
• ECOG performance status of 0-2.
• Histological, pathological, and/or cytological confirmation of either AdCC or SDC.
• Incurable local and/or regional recurrent and/or metastatic AdCC or SDC.
• Patients with AdCC can only participate in case of objective radiographic disease progression within three months before study enrollment and/or new or worsening disease-related symptoms that are not otherwise manageable during that same period.
• Measurable disease at baseline CT, defined by at least one lesion ≥2 cm (long axis).
• Positive [^{68}Ga]Ga-PSMA-11 PET/CT scan, defined by at least one lesion ≥1.5 cm (long axis) with a ligand SUV_{max} above liver SUV_{mean}.
• Sufficient bone marrow capacity as defined by:
  ▪ White blood cell count ≥2.5x10^9/L,
  ▪ Platelet count ≥100x10^9/L
  ▪ Hemoglobin ≥6 mmol/L
  ▪ Absolute neutrophil count ≥1.5x10^9/L
• Adequate liver function as defined by:
  ▪ Total bilirubin ≤1.5 x ULN. For patients known with Gilbert's Syndrome ≤3 x ULN is permitted.
  ▪ Alanine aminotransferase (ALT) and aspartate aminotransferase (AST) ≤3.0 × ULN or ≤5.0 × ULN for patients with liver metastases.
• Adequate kidney function as defined by:
  ▪ Serum creatinine ≤1.5 x ULN or creatinine clearance (CKD-EPI) ≥50 mL/min.

Exclusion criteria:

• Current pregnancy or breast-feeding.
• Known brain metastases or cranial epidural disease or intracardial metastases.
• Concurrent serious (as determined by the Principal Investigator) medical conditions, including, but not limited to, New York Heart Association class III or IV congestive heart failure, history of congenital prolonged QT syndrome, uncontrolled infection, active hepatitis B or C, or other significant comorbid conditions that in the opinion of the investigator would impair study participation or cooperation.
• Patients with urinary tract obstruction or marked hydronephrosis
• Less than 4 weeks since last myelosuppressive therapy or other radionuclide therapy.
• Concomitant cancer treatments.

Labeling and purification of PSMA-I&T with ^{177}Lu

$^{177}LuCl_3$ was obtained from ITG (Garching, Germany). GMP-grade PSMA-I&T was obtained from piCHEM (Raaba-Grambach, Austria). The radiolabeling of PSMA-I&T was performed on GRP synthesis module (Scintomics, Fürstenfeldbruck, Germany) using sterile and GMP-grade SC-105 kits. In brief, 4 mg gentisic acid and 250 µg PSMA-I&T peptide were dissolved in 500 µL WFI and added to the reaction vessel. After addition of the $^{177}LuCl_3$ in sodium acetate buffer and ascorbic acid the reaction was incubated at 100 °C for 20 min. After cooling down, the product was diluted to 16.5 ml with saline/DTPA to which 0.9 ml ethanol has been added. The radioactive solution was filtered through a 0.22 µm filter (Millex GV. Merck, Amsterdam, The Netherlands) and dispensed into a closed glass type I container. Microbiological monitoring in class C was performed during synthesis, filtration and dispensing. Assembling of the dispensing and filtration system was performed in a class A isolator with a class B airlock (in a class C background). The radiolabeled PSMA-I&T was measured for total radioactivity in an appropriately calibrated radioactive dose calibrator prior to injection. [^{177}Lu]Lu-PSMA was injected within 6 hours after radiolabeling.

Image acquisition and reconstruction

[^{68}Ga]Ga-PSMA-11 PET/CT

Patients underwent [^{68}Ga]Ga-PSMA-11 PET/CT within 4 weeks of radioligand therapy. Imaging was performed 60 ± 10 minutes post-injection on a Biograph mCT system (Siemens Healthineers, Erlangen, Germany) scanning cranium to trochanter major. For the pelvis region, data were acquired using 4 minutes per bed position, whereas 3 minutes per bed position were applied for the other regions. PET data were reconstructed using ordinary Poisson ordered-subset expectation maximization with time-of-flight modeling (OP-OSEM-TOF) with 2 iterations and 21 subsets, matrix size of 400 (resulting in a cuboid-shape voxel size of approximately 8.0 (2.0 x 2.0 x 2.0) mm^3) and a smoothing Gaussian filter of 3 mm. The estimated reconstructed PET spatial resolution expressed as full width at half maximum (FWHM) was 6.3 mm. A low dose CT was performed (average dose length product (DLP) of 291 mGy·cm) and the CT data were reconstructed using 3.0 mm slice thickness and kernel B19f; the reconstructed transverse CT images had a voxel size of 1.0 x 1.0 x 3.0 mm^3. For all PET images, standard corrections for CT-based attenuation, scatter, decay and dead-time were performed.

[^{177}Lu]Lu-PSMA SPECT/CT

SPECT/CT and planar imaging was performed at 1, 24, 48, 72 and 168 hours after administration of [^{177}Lu]Lu-PSMA-I&T on either a Symbia T16 or Symbia Intevo Bold system (Siemens Healthineers, Erlangen, Germany). SPECT/CT scans were acquired at two body regions: head/neck until mid-thorax and mid-thorax until abdomen (64 projections per detector, time per projection of 14 s, a 20% photon energy window at 208 keV, and dual-energy window for Compton scattering. SPECT data were reconstructed using OSEM reconstruction (Flash 3D with collimator detector response) using 4 iterations and 8 subsets, matrix size of 128 (resulting in a cuboid-shape voxel size of 4.8 mm^3) and a smoothing Gaussian filter of 8.4 mm. The estimated reconstructed SPECT spatial resolution was 15 mm (FWHM). A low dose CT was performed (average DLP of 130 mGy·cm) and the data were reconstructed using B31s kernel and 3.0 mm slice thickness resulting in a voxel size of 1.0 x 1.0 x 3.0 mm^3. Of note, the SPECT image reconstruction approach takes into account corrections for scatter, CT-based attenuation and dead-time.

Supplementary Tables

Table S1: Study assessments table.

Week	Blood analysis	ECG	ctDNA	OPD visit	^{68}Ga-PSMA PET/CT	^{18}FDG PET/CT	^{177}Lu-PSMA-I&T treatment	SPECT/CT	CT	Question-naires	Optional biopsy
Screening	X	X	X	X	X	X			X		
Baseline										X	X
0	X						X	X			
1	X							X			
2	X	X	X	X							
4	X		X	X							
6	X			X			X	X		X	
8	X			X							
10	X		X	X	X	X			X		X
12	X			X			X	X		X	
15	X			X							
18	X			X			X	X		X	
21	X			X							
24	X		X	X							
+3	X		X	X	X				X	X	
+6	X		X	X					X	X	
At PD	X		X	X					X	X	X

Abbreviations: ^{177}Lu: Lutetium-177; ^{18}FDG: 2-deoxy-2-[^{18}F]fluoro-D-glucose; ^{68}Ga: gallium-68; CT: computed tomography; ctDNA: circulating tumor DNA; ECG: electrocardiogram; OPD: outpatient department; PD: progressive disease; PET: positron emission tomography; PSMA: prostate-specific membrane antigen; SPECT: single-photon emission computed tomography.

Table S2: All adverse events (any cause).

Adverse event	Any Grade<br>No. of pts (%)	Grade 1<br>No. of pts (%)	Grade 2<br>No. of pts (%)	Grade 3<br>No. of pts (%)	Grade ≥4<br>No. of pts (%)
Dry mouth	10 (83%)	7 (58%)	3 (25%)	0	N/A
Fatigue	10 (83%)	7 (58%)	3 (25%)	0	N/A
Nausea	9 (75%)	6 (50%)	3 (25%)	0	N/A
Anemia	7 (58%)	7 (58%)	0	0	0
Hyponatremia	4 (33%)	2 (17%)	1 (8%)	1 (8%)	0
Lymphocyte count decreased	4 (33%)	2 (17%)	1 (8%)	1 (8%)	0
Neutrophil count decreased	4 (33%)	2 (17%)	1 (8%)	1 (8%)	0
Constipation	4 (33%)	3 (25%)	1 (8%)	0	0
Dyspnea	4 (33%)	3 (25%)	1 (8%)	0	0
Aspartate aminotransferase increased	3 (25%)	1 (8%)	1 (8%)	1 (8%)	0
Dysgeusia	3 (25%)	3 (25%)	0	N/A	N/A
Headache	3 (25%)	3 (25%)	0	0	
Hypokalemia	3 (25%)	3 (25%)	0	0	0
Vomiting	3 (25%)	3 (25%)	0	0	0
Paresthesia	2 (17%)	1 (8%)	0	1 (8%)	N/A
Anorexia	2 (17%)	0	2 (17%)	0	0
Tumor pain	2 (17%)	1 (8%)	1 (8%)	0	N/A
Cough	2 (17%)	2 (17%)	0	0	N/A
Diarrhea	2 (17%)	2 (17%)	0	0	0
Platelet count decreased	2 (17%)	2 (17%)	0	0	0
Chronic kidney disease	1 (8%)	0	1 (8%)	0	0
Dyspepsia	1 (8%)	0	1 (8%)	0	N/A
Edema limbs	1 (8%)	0	1 (8%)	0	N/A
Fall	1 (8%)	0	1 (8%)	0	N/A
Hypertension	1 (8%)	0	1 (8%)	0	0
Urinary tract infection	1 (8%)		1 (8%)	0	0
Urinary urgency	1 (8%)	0	1 (8%)	N/A	N/A
Anxiety	1 (8%)	1 (8%)	0	0	0
Aspiration	1 (8%)	1 (8%)	0	0	0
Back pain	1 (8%)	1 (8%)	0	0	N/A
Bladder spasm	1 (8%)	1 (8%)	0	0	N/A
Bone pain	1 (8%)	1 (8%)	0	0	N/A
Chest wall pain	1 (8%)	1 (8%)	0	0	N/A
Dizziness	1 (8%)	1 (8%)	0	0	N/A
Epistaxis	1 (8%)	1 (8%)	0	0	0
Fever	1 (8%)	1 (8%)	0	0	0
Gastrointestinal pain	1 (8%)	1 (8%)	0	0	N/A
Hearing impaired	1 (8%)	1 (8%)	0	0	0

Hematoma	1 (8%)	1 (8%)	0	0	0
Hematuria	1 (8%)	1 (8%)	0	0	0
Hiccups	1 (8%)	1 (8%)	0	0	N/A
Hyperkalemia	1 (8%)	1 (8%)	0	0	0
Blood bilirubin increased	1 (8%)	1 (8%)	0	0	0
Myalgia	1 (8%)	1 (8%)	0	0	N/A
Non-cardiac chest pain	1 (8%)	1 (8%)	0	0	N/A
Oral pain	1 (8%)	1 (8%)	0	0	N/A
Pain	1 (8%)	1 (8%)	0	0	N/A
Pain in extremity	1 (8%)	1 (8%)	0	0	N/A
Pharyngolaryngeal pain	1 (8%)	1 (8%)	0	0	N/A
Productive cough	1 (8%)	1 (8%)	0	0	N/A
Stomach pain	1 (8%)	1 (8%)	0	0	N/A
Watering eyes	1 (8%)	1 (8%)	0	0	0

Adverse events were graded according to National Cancer Institute Common Terminology Criteria for Adverse Events (version 5.0). Patients were counted once at the highest grade for each adverse event. Abbreviations: N/A: not applicable

Table S3: Index tumor lesions [^{68}Ga]Ga-PSMA-11 PET/CT SUV values, tumor-absorbed doses per unit administered activity and cumulative dose (Gy/GBq or Gy), and tumor volumes before and after ^{177}Lu-PSMA therapy.

Pt No.	Histological subtype	Total injected ^{177}Lu-PSMA activity (GBq)	Tumor site	Liver SUV_{mean} ^{68}Ga-PSMA PET	Tumor SUV_{mean} ^{68}Ga-PSMA PET	Tumor-to-liver SUV_{mean} ratio	Tumor SUV_{max} ^{68}Ga-PSMA PET	Dose (Gy/GBq)	Cumulative dose (Gy)	Tumor volume pre-therapy (cm^3)	Tumor volume post-therapy (cm^3)	Tumor volume change
1	AdCC	29.52	Lung	3.25	2.04	0.63	6.30	0.06	1.89	104.7	94.5	-9.7%
			Lung		2.38	0.73	5.39	0.06	1.83	82.2	76.7	-6.7%
			Lung		2.97	0.91	5.56	0.06	1.89	11.7	10.7	-8.9%
			Pleura		2.94	0.90	5.14	0.04	1.30	67.9	63.8	-6.0%
			Lung		3.13	0.96	5.70	0.08	2.33	19.0	14.6	-23.4%
			Pleura		2.93	0.90	7.86	0.06	1.89	280.1	270.3	-3.5%
			Pleura		2.96	0.91	4.87	0.03	0.80	2.7	2.4	-12.5%
			Pleura		2.58	0.79	4.92	0.03	0.97	4.0	3.3	-16.5%
			Pleura		2.86	0.88	4.93	0.03	1.00	10.6	10.5	-1.1%
			Pleura		2.84	0.87	4.07	0.06	1.01	33.4	30.2	+9.5%
			Lymph node		1.55	0.48	1.72	0.04	1.09	5.4	5.5	+2.0%
2	AdCC	14.80	Lung	5.85	2.94	0.50	5.16	0.02	0.31	6.3	12.1	+93.6%
			Lung		4.18	0.71	6.54	0.03	0.41	17.9	33.0	+84.1%
			Lymph node		3.61	0.62	4.29	0.02	0.28	0.7	1.7	+123.0%
			Bone		8.37	1.43	15.30	0.06	0.84	3.8	12.0	+218.0%
3	AdCC	29.63	Lung	4.25	4.03	0.95	7.12	0.34	9.96	6.3	9.1	+45.8%
			Lung		4.52	1.06	9.07	0.37	10.93	20.0	30.9	+54.4%
			Lung		7.84	1.84	13.80	0.45	13.21	54.7	157.5	+188.1%
			Lung		5.56	1.31	11.08	0.63	18.67	31.4	45.6	+45.3%
			Lung		4.25	1.00	8.86	0.26	7.76	7.8	11.8	+51.4%
			Lung		4.45	1.05	7.08	0.42	12.59	9.4	13.8	+47.1%
			Lung		3.80	0.89	5.74	0.19	5.75	9.2	13.5	+46.5%
			Lung		4.86	1.14	8.21	0.27	7.94	22.4	30.8	+37.3%
			Liver		5.55	1.31	6.71	0.11	3.17	1.1	1.4	+29.4%

4	AdCC	14.80	Local recurrence	7.87	7.29	0.93	16.77	0.10	1.54	2.2	3.7	+71.9%
5	AdCC	29.11	Lung	6.07	2.49	0.41	4.69	0.11	3.26	2.5	2.3	-9.6%
			Lung		2.78	0.46	5.13	0.07	2.13	3.6	3.1	-13.7%
			Lung		2.25	0.37	3.68	0.06	1.81	3.7	2.9	-23.8%
			Liver		6.60	1.09	12.67	0.02	0.47	160.5	155.6	-3.1%
			Liver		8.53	1.41	13.73	0.03	0.84	135.0	136.9	+1.4%
			Liver		9.51	1.57	13.20	0.03	0.79	87.2	94.0	+7.8%
6	AdCC	14.83	Lung	4.16	2.81	0.68	4.25	0.01	0.26	4.5	5.7	+28.4%
			Lung		3.21	0.77	4.50	0.02	0.31	3.7	4.5	+23.4%
			Lung		5.14	1.24	9.29	0.05	0.68	91.9	92.7	+0.9%
			Lung		2.42	0.58	4.09	0.01	0.21	1.8	2.3	+27.1%
			Lung		4.14	1.00	9.17	0.02	0.24	7.3	11.2	+53.4%
			Pleura		4.42	1.06	7.75	0.001	0.02	8.9	11.9	+33.9%
			Pleura		3.50	0.84	7.46	0.03	0.37	4.7	7.4	+57.4%
			Pleura		3.96	0.95	7.54	0.05	0.74	9.4	10.4	+10.3%
7	AdCC	29.76	Lung	6.62	3.66	0.55	5.47	0.12	3.51	8.5	13.1	+54.9%
			Lung		6.01	0.91	8.12	0.20	6.07	4.5	7.2	+62.3%
			Lung		2.63	0.40	3.82	0.05	1.58	3.7	4.4	+17.7%
			Lung		3.22	0.49	5.36	0.09	2.65	1.9	2.0	+5.2%
			Lung		6.08	0.92	7.93	0.17	4.94	5.9	7.1	+19.5%
8	AdCC	7.39	Lung	4.09	4.27	1.04	5.28	0.39	2.87	0.3	0.3	+6.3%
			Lung		5.00	1.22	7.08	0.21	1.54	1.3	1.1	-13.5%
			Lung		5.58	1.36	8.68	0.14	1.06	1.3	1.1	-13.8%
			Pleura		2.64	0.65	4.51	0.02	0.18	0.7	0.8	+15.1%
			Pleura		3.39	0.83	4.03	0.05	0.35	0.9	0.9	+2.3%
			Lung		6.63	1.62	10.50	0.08	0.55	8.6	7.8	-9.8%
			Bone		6.85	1.67	11.15	0.12	0.86	6.9	5.3	-23.1%
			Bone		10.73	2.62	18.77	0.25	1.85	10.7	15.7	+46.1%
			Bone		8.54	2.09	18.09	0.34	2.51	8.5	18.8	+120.2%
9	AdCC	29.78	Pleura	3.32	4.54	1.37	8.61	0.03	0.92	6.3	5.2	-17.3%
			Pleura		3.89	1.17	5.61	0.01	0.41	0.6	0.9	+58.6%
			Lung		4.44	1.34	7.76	0.27	7.87	8.7	13.4	+54.8%
			Lung		5.39	1.62	9.70	5	7.42	26.2	26.2	+0.2%
			Pleura		4.80	1.45	9.60	0.02	0.50	1.5	3.2	+120.7%

			Pleura		3.63	1.09	5.78	0.05	1.54	3.4	6.7	+97.9%
			Liver		6.52	1.96	12.92	0.08	2.34	5.9	16.9	+186.4%
			Lymph node		4.33	1.30	7.00	0.02	0.71	2.1	3.2	+50.0%
10	AdCC	29.49	LR	7.75	7.32	0.94	14.92	0.40	11.74	44.6	87.0	+95.1%
			Lung		4.47	0.58	8.17	0.57	15.51	1.3	2.2	+67.2%
			Pleura		3.85	0.50	6.26	0.24	6.99	0.6	1.3	+130.9%
			Lung		3.03	0.39	4.73	0.39	11.47	0.8	1.4	+84.6%
			Thoracic wall		2.58	0.33	4.29	0.11	3.24	2.3	3.7	+60.3%
11	SDC	14.99	Lymph node	4.72	3.79	0.80	5.18	0.04	0.62	2.3	2.2	-4.8%
			Lymph node		3.72	0.79	5.03	0.03	0.42	1.5	1.8	+22.0%
			Lymph node		3.42	0.72	4.57	0.01	0.08	1.7	1.9	+10.4%
			Liver		7.29	1.54	13.45	0.27	4.09	6.8	25.4	+270.6%
			Liver		7.78	1.65	11.93	0.12	1.83	182.4	233.4	+28.0%
			Liver		7.59	1.61	13.42	0.34	5.16	8.2	39.9	+384.7%
			Bone		9.06	1.92	17.62	0.29	4.27	7.2	3.0	-57.7%
			Bone		7.16	1.52	16.78	0.16	2.41	26.4	36.3	+37.5%
			Mean	5.27	4.71	1.04	5.15	0.14	3.28	23.6	28.4	+43.3%
			SD	1.66	2.04	0.45	4.03	0.15	4.08	47.2	51.1	+71.3%
			Median	4.72	4.18	0.95	7.08	0.07	1.58	6.3	8.4	+27.5%
			Minimum	3.25	1.55	0.33	1.72	0.001	0.02	0.3	0.3	-57.7%
			Maximum	7.87	10.73	2.62	18.77	0.63	18.67	280.1	270.3	+384.7%

Abbreviations: ^{177}Lu: lutetium-177; ^{68}Ga: gallium-68; AdCC: adenoid cystic carcinoma; SD: standard deviation; PET: positron emission tomography; Pt No.: patient number; SDC: salivary duct carcinoma; SUV_{max}: maximum standardized uptake value; SUV_{mean}: mean standardized uptake value.

Table S4: Absorbed dose in organs at risk per patient per unit administered activity and cumulative dose (Gy/GBq or Gy).

Pt No.	Histological subtype	Total injected ^{177}Lu-PSMA activity (GBq)	Kidneys (Gy/GBq)	Kidneys (Gy)	Salivary glands (Gy/GBq)	Salivary glands (Gy)	Bone marrow (Gy/GBq)	Bone marrow (Gy)
1	AdCC	29.52	0.42	12.31	0.42	12.45	0.007	0.198
2	AdCC	14.80	0.73	10.81	0.38	5.66	0.009	0.135
3	AdCC	29.63	0.83	24.47	1.15	34.07	0.008	0.121
4	AdCC	14.80	0.89	13.11	1.03	15.25	0.010	0.294
5	AdCC	29.11	0.72	20.96	0.98	28.40	0.007	0.110
6	AdCC	14.83	1.10	16.31	0.33	4.92	0.008	0.227
7	AdCC	29.76	1.31	38.95	1.02	30.37	0.006	0.041
8	AdCC	7.39	0.67	4.97	0.69	5.10	0.007	0.199
9	AdCC	29.78	0.52	15.41	0.27	7.91	0.003	0.102
10	AdCC	29.49	1.34	39.51	0.60	17.59	0.007	0.099
11	SDC	14.99	0.53	7.96	1.28	19.20	0.006	0.169

Abbreviations: ^{177}Lu, lutetium-177; AdCC: adenoid cystic carcinoma; Pt No.: patient number; SDC: salivary duct carcinoma.

Supplementary Figures

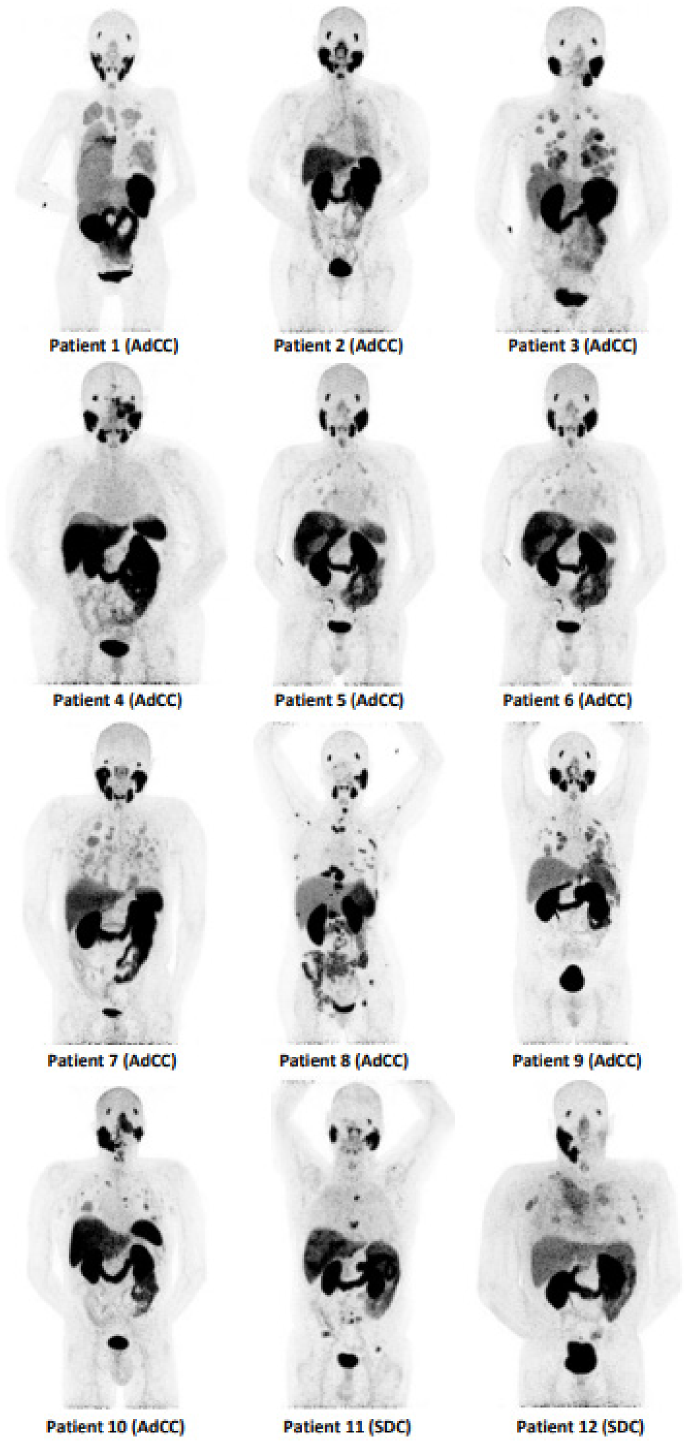

Figure S1: [^{68}Ga]Ga-PSMA-11 PET/CT maximum intensity projections before [^{177}Lu]Lu-PSMA-I&T treatment. Abbreviations: ^{177}Lu: Lutetium-177; ^{68}Ga: gallium-68; AdCC: adenoid cystic carcinoma; CT: computed tomography; PET: positron emission tomography; PSMA: prostate-specific membrane antigen; SDC: salivary duct carcinoma.

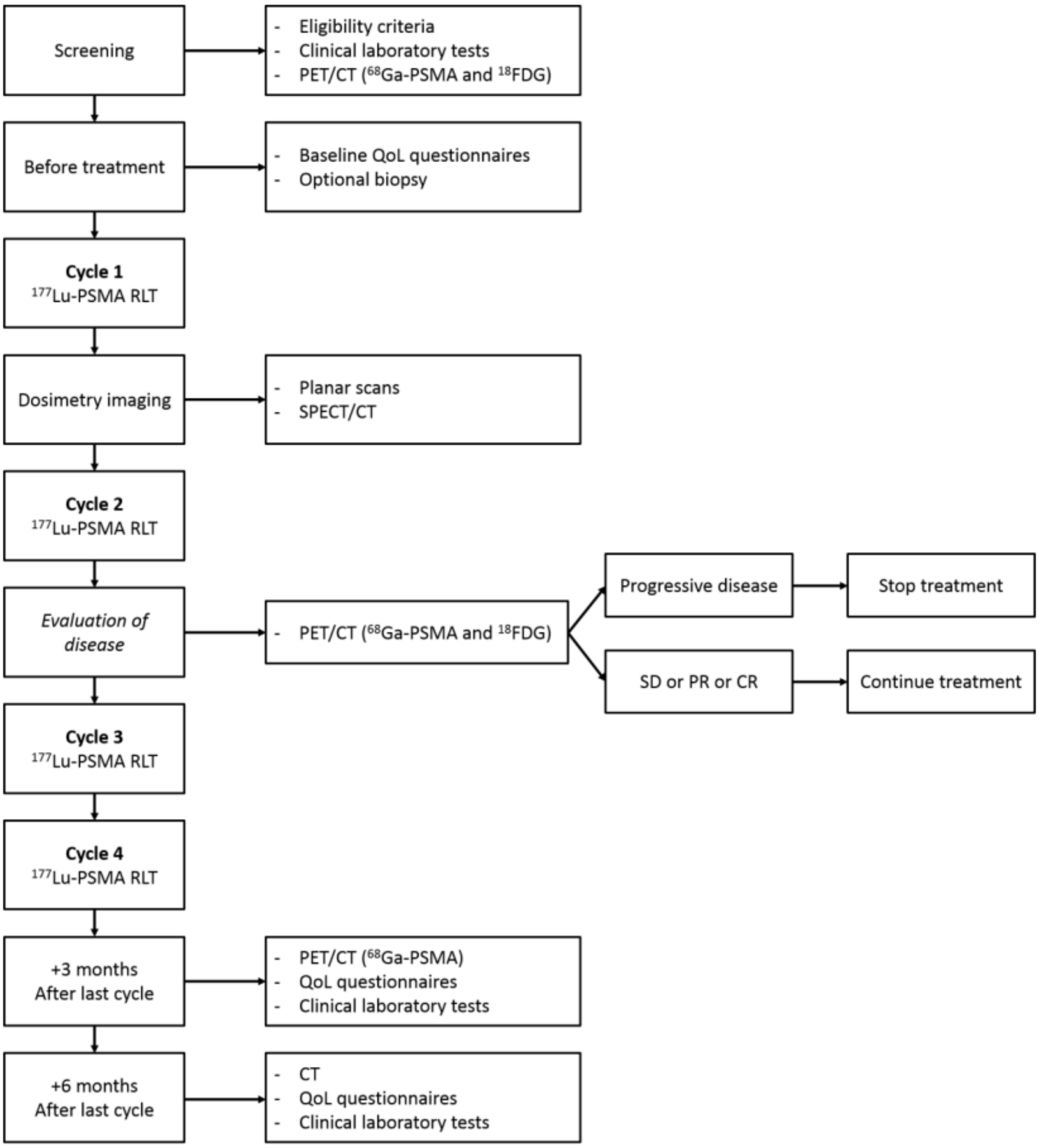

Figure S2: Study flowchart. Abbreviations: ^{177}Lu: Lutetium-177; ^{18}FDG: [^{18}F]Fluorodeoxyglucose; ^{68}Ga: gallium-68; CR: complete response; CT: computed tomography; PD: progressive disease; PET: positron emission tomography; PR: partial response; QoL: quality of life; PSMA: prostate-specific membrane antigen; RLT: radioligand therapy; SD: stable disease; SPECT: single-photon emission computed tomography.

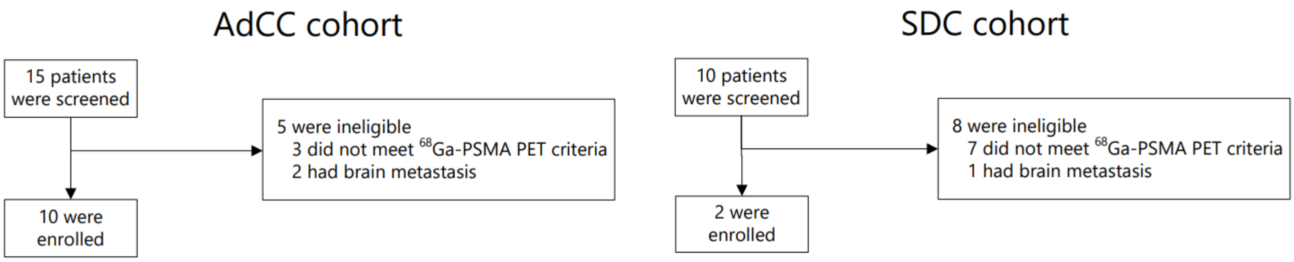

Figure S3: Trial profile of the AdCC and the SDC cohort. Abbreviations: ^{68}Ga: gallium-68; AdCC: adenoid cystic carcinoma; PET: positron emission tomography; PSMA: prostate-specific membrane antigen; SDC: salivary duct carcinoma.

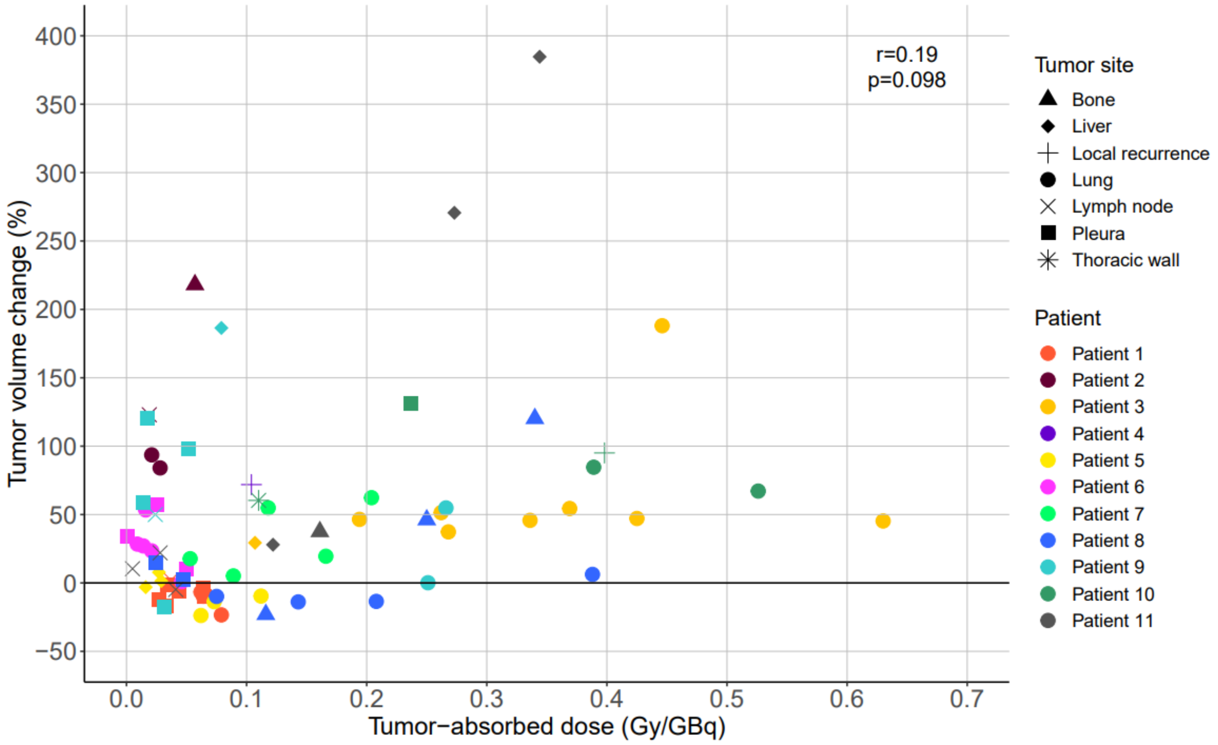

Figure S4: Scatter plot displaying the correlation between the absorbed radiation dose per index tumor lesion and the tumor volume change after [^{177}Lu]Lu-PSMA-I&T therapy compared to baseline. Abbreviations: ^{177}Lu: lutetium-177; PSMA: prostate-specific membrane antigen.

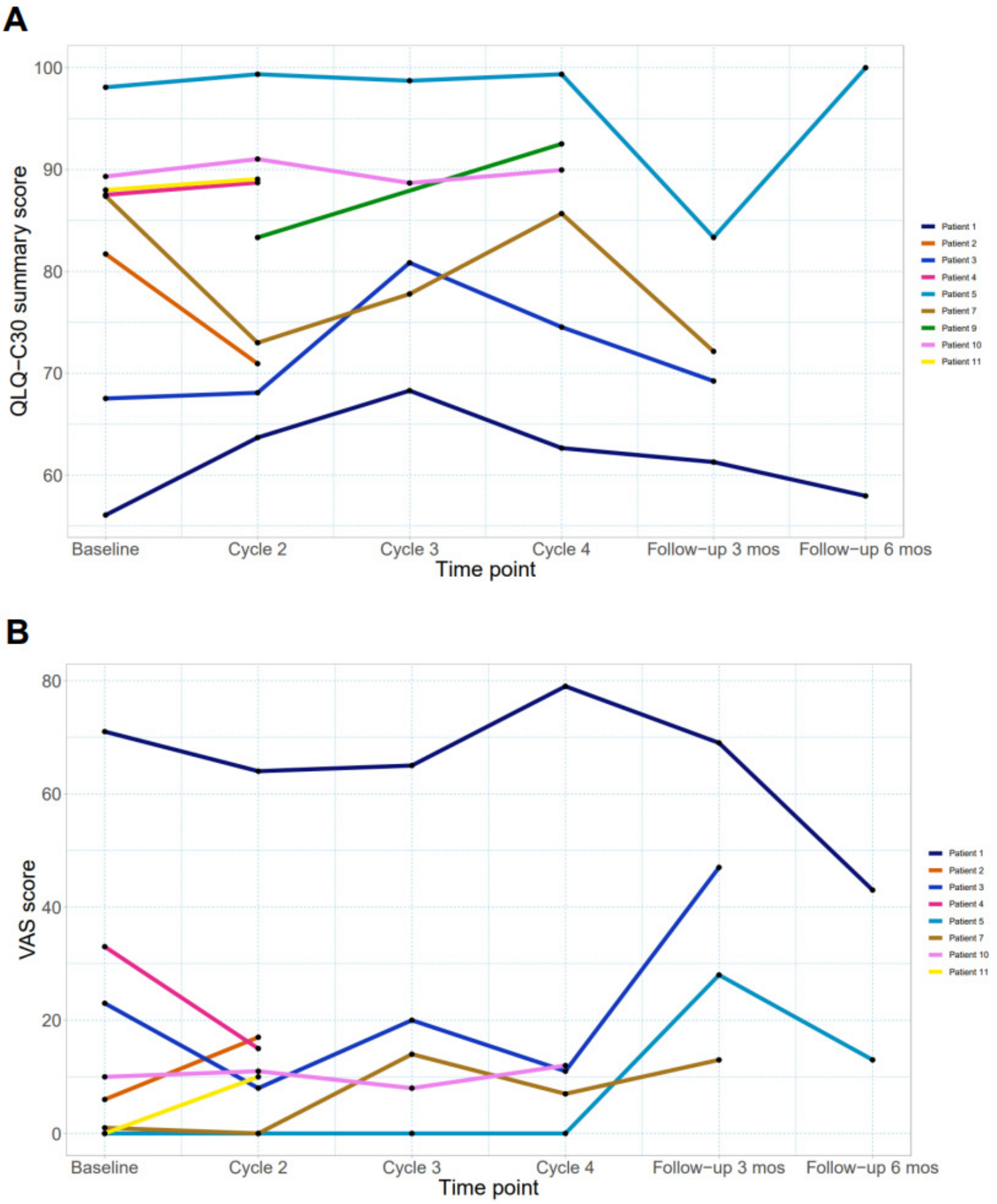

Figure S5: Individual patient-reported outcome measure scores. (A) EORTC-QLQ-C30 summary score. **(B)** VAS score. Patients with ≥2 responses are displayed. Abbreviations: EORTC: European Organization for the Research and Treatment of Cancer; mos: months; QLQ-C30: Quality of Life Questionnaire-Core 30; VAS, visual analogue scale.
